# Mutated *CCDC51* Coding for a Mitochondrial Protein, MITOK Is a Candidate Gene Defect for Autosomal Recessive Rod-Cone Dystrophy

**DOI:** 10.3390/ijms22157875

**Published:** 2021-07-23

**Authors:** Christina Zeitz, Cécile Méjécase, Christelle Michiels, Christel Condroyer, Juliette Wohlschlegel, Marine Foussard, Aline Antonio, Vanessa Démontant, Lisa Emmenegger, Audrey Schalk, Marion Neuillé, Elise Orhan, Sébastien Augustin, Crystel Bonnet, Amrit Estivalet, Frédéric Blond, Steven Blanchard, Camille Andrieu, Sandra Chantot-Bastaraud, Thierry Léveillard, Saddek Mohand-Saïd, José-Alain Sahel, Isabelle Audo

**Affiliations:** 1 Sorbonne Université, INSERM, CNRS, Institut de la Vision, 75012 Paris, France; cecile.mejecase@hotmail.fr (C.M.); christelle.michiels@inserm.fr (C.M.); christel.condroyer@inserm.fr (C.C.); jwohls@uw.edu (J.W.); foussard.marine@gmail.com (M.F.); aline.antonio@inserm.fr (A.A.); vanessa.demontant@aphp.fr (V.D.); lisa.emmenegger@mdc-berlin.de (L.E.); audrey.schalk@chru-strasbourg.fr (A.S.); marion.neuille@gmail.com (M.N.); elise.orhan@yahoo.fr (E.O.); sebastien.augustin@inserm.fr (S.A.); crystel.bonnet@inserm.fr (C.B.); amrit.estivalet@inserm.fr (A.E.); frederic.blond@inserm.fr (F.B.); thierry.leveillard@inserm.fr (T.L.); saddekms@gmail.com (S.M.-S.); j.sahel@gmail.com (J.-A.S.); 2 CHNO des Quinze-Vingts, INSERM-DHOS CIC 1423, 75012 Paris, France; candrieu@15-20.fr; 3 Unité de Génétique et Physiologie de l’Audition, Institut Pasteur, 75015 Paris, France; 4 INSERM, Institut de l’Audition, Institut Pasteur, 75012 Paris, France; 5 IntegraGen SA, Genopole, Campus, 91000 Evry, France; steven.blanchard@integragen.com; 6 Fondation Ophtalmologique Adolphe de Rothschild, 75019 Paris, France; 7 APHP, Hôpital Armand-Trousseau, Département de Génétique, UF de Génétique Chromosomique, 75012 Paris, France; sandra.chantot-bastaraud@aphp.fr; 8 Academie des Sciences, Institut de France, 75006 Paris, France; 9 Department of Ophthalmology, The University of Pittsburgh School of Medicine, Pittsburgh, PA 15213, USA; 10 Institute of Ophthalmology, University College of London, London EC1V 9EL, UK

**Keywords:** rod-cone dystrophy, retinitis pigmentosa, candidate gene, *CCDC51*, MITOK, mitochondrial protein, inner segments, retina

## Abstract

The purpose of this work was to identify the gene defect underlying a relatively mild rod-cone dystrophy (RCD), lacking disease-causing variants in known genes implicated in inherited retinal disorders (IRD), and provide transcriptomic and immunolocalization data to highlight the best candidate. The DNA of the female patient originating from a consanguineous family revealed no large duplication or deletion, but several large homozygous regions. In one of these, a homozygous frameshift variant, c.244_246delins17 p.(Trp82Valfs*4); predicted to lead to a nonfunctional protein, was identified in *CCDC51*. *CCDC51* encodes the mitochondrial coiled-coil domain containing 51 protein, also called MITOK. MITOK ablation causes mitochondrial dysfunction. Here we show for the first time that CCDC51/MITOK localizes in the retina and more specifically in the inner segments of the photoreceptors, well known to contain mitochondria. Mitochondrial proteins have previously been implicated in IRD, although usually in association with syndromic disease, unlike our present case. Together, our findings add another ultra-rare mutation implicated in non-syndromic IRD, whose pathogenic mechanism in the retina needs to be further elucidated.

## 1. Introduction

Non-syndromic rod-cone dystrophy (RCD), also reported as retinitis pigmentosa, is a progressive retinal disease characterized by night blindness, progressive visual field constriction, and, in severe cases, total blindness with central vision loss, with a worldwide prevalence of 1 in 4000 [1]. This heterogeneous disease is inherited as an autosomal recessive, autosomal dominant, or X-linked trait [2]. Mutations in eighty different genes have been reported in non-syndromic RCDs (https://sph.uth.edu/retnet/sum-dis.htm, accessed on 9 April 2021). Recent studies performing targeted next generation sequencing (NGS), whole exome sequencing (WES), and whole genome sequencing (WGS) report a mutation detection rate in RCD or in general in inherited retinal disorder (IRD) cohorts between ~50 and 85% [3,4,5,6,7]. The remaining unsolved cases may harbor mutations in intronic or regulatory sequences, may initially be undetected due to poorly covered DNA regions, or represent copy number variants (CNVs) missed by the chosen sequencing method. In addition, novel gene defects accounting for the remaining patient population are most likely ultra-rare, most of the major defects being already identified and may therefore lack population replication [8,9,10]. As previously shown, initial targeted NGS and subsequent WES on patients lacking mutations in known genes underlying IRD is an efficient strategy to identify novel gene defects [10,11,12,13,14,15,16,17]. The purpose of the work herein was to identify a disease-associated gene involved in a sporadic RCD case, previously excluded for a known gene defect by targeted NGS.

## 2. Results

### 2.1. A Sporadic Case with Mild arRCD

**Figure 1:**
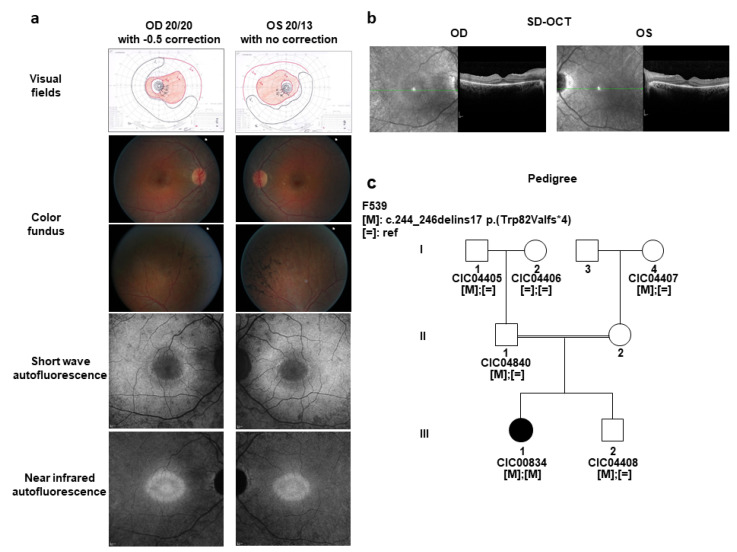
Clinical data and pedigree of a patient from a consanguineous family with rod-cone dystrophy. (**a**) Kinetic visual field show an annular scotoma for both eyes with relatively conserved peripheral isopters. Fundus color photographs show normal optic discs and retinal vessels with pigmentary changes in the periphery. Short- and near infrared wavelength fundus autofluorescence show a perifoveal ring of increased autofluorescence. (**b**) Spectral domain optical coherence tomography (SD-OCT) reveals well preserved outer retinal bands at the macula with outer retinal thinning in the periphery. (**c**) The affected girl (III.1) is homozygous for c.244_246delins17 p.(Trp82Valfs*4) [M] in CCDC51. The unaffected brother (III.2), the unaffected father (II.1), the unaffected paternal grandfather (I.1), and the unaffected maternal grandmother (I.4) are heterozygous for this variant, while the unaffected paternal grandmother (I.2) has no variant [=]. Circles represent female, squares represent male. Empty symbols indicate unaffected individuals, and the filled symbol affected individuals.

A 25-year-old affected woman from a consanguineous union from Morocco was diagnosed with mild RCD (Figure 1). She had no relevant medical history. She maintained relatively good central vision with a best corrected visual acuity (BCVA) of 20/20 with −0.5 correction for the right and 20/13 with no correction for the left eye. Kinetic visual fields showed an annular scotoma on both eyes with relatively well preserved peripheral isopters (Figure 1a). Full-field electroretinogram (ff-ERG) revealed only residual photopic responses in keeping with generalized rod-cone dysfunction. Fundus color examination revealed normal optic discs and retinal vessels with pigmentary changes in the periphery (Figure 1a). Short- and near infrared wavelength fundus autofluorescence revealed a perifoveal ring of increased autofluorescence which corresponded to preserved outer retina on spectral domain optical coherence tomography (SD-OCT) (Figure 1b).

### 2.2. Identification of a Novel Candidate Gene, CCDC51 Underlying Mild arRCD

After exclusion by targeted NGS of disease-causing variants in 123 genes previously associated with RCD [11], array comparative genomic hybridization (CGH) the index patient (CIC00834, III.1, Figure 1) revealed no large putative disease-causing CNVs. However, this approach identified fifteen relatively large (>2 Mb) homozygous regions of particular interest since previous studies have shown that large homozygous regions often harbor recessive disease-causing variants in families of consanguineous origin [18].

**Figure 2:**
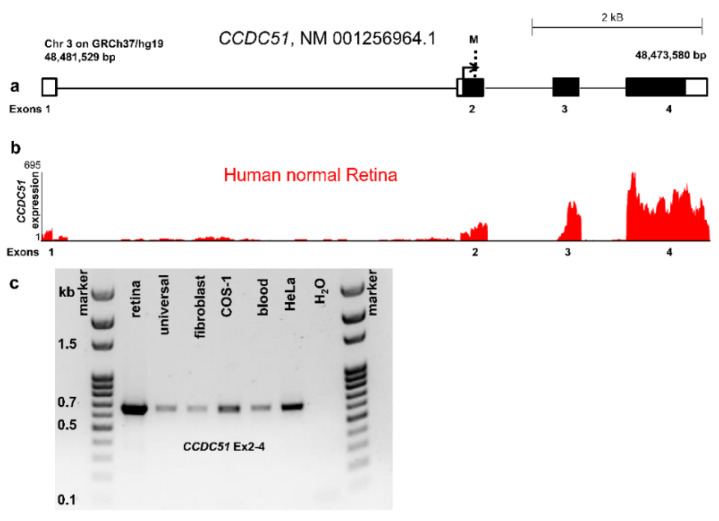
Schematic drawing of the genomic structure and expression of *CCDC51*. (**a**) *CCDC51* is located on chromosome 3 and exons 2–4 code for a 411 amino acid protein. Unfilled and filled boxes represent untranslated and translated regions, respectively. The arrow depicts the translation start. M represents the identified mutation. (**b**) The four exons of *CCDC51* are expressed in normal human retina (red) [20]. (**c**) *CCDC51* RT-PCR encompassing exons 2–4 revealed the presence of the transcript in all tested tissues and cells including human retina, human universal tissues control cDNA, human fibroblasts, human blood, COS-1, and HeLa cells shown on an agarose gel with an expected size of ~660 bp.

Among these homozygous regions, the four largest had sizes of 25, 18, 20, and 13 Mb and mapped to chromosomes 2, 3, 12, and 15, respectively. Subsequently, WES, applied on the genomic DNA of the affected girl (CIC00834, III.1, Figure 1), her unaffected father (CIC04840, II-1, Figure 1), and her unaffected brother (CIC04408, III-2, Figure 1c) identified 37,452 single nucleotide variants (SNVs) and 2979 insertions or deletions (InDels). Stringent filtering was applied to identify the disease-causing variants as described in the Materials and Methods section. Due to the reported consanguinity, we prioritized homozygous variants present in the affected girl, but heterozygous in the unaffected father and heterozygous or absent in the unaffected brother. In addition, compound heterozygous variants present in the affected girl, heterozygous in the unaffected father and heterozygous or absent in the unaffected brother were also considered [19]. Applying this stringent filtering, only the homozygous c.244_246delins17 p.(Trp82Valfs*4) InDel in exon 2 of *CCDC51* (NM_001256964.2) (will be submitted to https://databases.lovd.nl/shared/genes/CCDC51, accessed on 9 April 2021) remained on the list (Figure 1c and Figure 2, Table 1). The 3 nucleotides 5′-TGG-3′ at positions 244 to 246 were deleted and replaced by 17 nucleotides 5′-GTGGAGATATGAAGATA-3′ in exon 2. This is predicted to lead to a shift in the coding frame and a shorter form of the protein or nonsense-mediated mRNA decay. The variant was validated in the index patient as well as in all available family members by Sanger sequencing and co-segregated with the phenotype in the three-generation family following an autosomal recessive mode of inheritance (Figure 1c).

According to the American College of Medical Genetics and Genomics standards, the variant was classified as pathogenic (1a) (PVS1, PVS4, PM2, and PM3) [21,22]. This probably disease-causing variant is rare and is not reported in current variant databases including gnomAD. *CCDC51*, mapping to chromosome 3 (48,473,580–48,481,529 bps on GRCh37/hg19) contains 4 exons of which exons 2–4 are coding (Figure 2a). Interestingly, the 18 Mb homozygous region on chromosome 3 (chr3:31,849 818–50,174,572) identified in the index patient by array CGH contains this *CCDC51* variant, reinforcing this gene defect as a candidate to be implicated in autosomal recessive RCD in this consanguineous family. Neither one of >1.000 other cases from our French RCD cohort investigated using Sanger sequencing nor >2.000 other cases from cohorts of our worldwide collaborators revealed other convincing biallelic disease-causing variants in *CCDC51*. In the studied cohort, three cases harbored a monoallelic putative pathogenic variant in *CCDC51* (Table S1). However, quantitative PCR experiments using primers covering all exons (Table S2) did not reveal CNVs in the four coding exons of *CCDC51*. The genomic region of these patients needs to be investigated to evaluate if variants in introns or in regulatory regions contribute to the disease. Together, screening results of *CCDC51* revealed that this gene defect is an ultra-rare cause of autosomal recessive RCD as it has been previously found for other novel gene defects underlying IRD [8,9,10]. Similarly, rare loss-of-function mutations in another mitochondrial gene, *IDH3B*, led to non-syndromic RCD in three families [23,24] and was mentioned to be mutated in a patient with an unspecified syndrome [25].

### 2.3. Function and Pathogenic Mechanism of the Mitochondrial Protein MITOK Encoded by CCDC51

CCDC51 encodes the coiled-coil domain-containing protein 51. Exons 2–4 of *CCDC51* code for a mitochondrial protein CCDC51 [26], also called MITOK [27] with 411 amino acids (aa) with a predicted N-terminal mitochondrial targeting sequence, a coiled-coil domain (aa 111–173), and two transmembrane helical domains (aa TM1 202–222 and TM2 387–407). Recent findings have shown that CCDC51 is an inner membrane mitochondrial 45 kDa protein, of which both the N- and C-termini are exposed toward the internal matrix, while the region between the two transmembrane domains (aa 223–386) is in the intermembrane space of the mitochondria. CCDC51, alias MITOK, presents the pore-forming subunit of a mitoK (ATP) channel [24], while MITOSUR represents the ATP-binding subunit. Genetic ablation of MITOK causes instability in the mitochondrial membrane potential, widening the intracristae space and decreasing oxidative phosphorylation in vitro [27]. Since the homozygous variant, c.244_246delins17 p.(Trp82Valfs*4) InDel in exon 2 of *CCDC51* identified herein causes a frameshift at the N-terminus of CCDC51 and a shorter form of the protein, with only 82 amino acids lacking mitochondrial and transmembrane domains or leading to nonsense-mediated mRNA decay, the mutated protein of the patient is also predicted to be nonfunctional leading to a mitochondrial defect in the retina. This, together with decreased oxidative phosphorylation in the retina, may be associated with photoreceptor degeneration and RCD. A mouse model lacking CCDC51 showed suppression of cardioprotection [27]. Similarly, other studies strengthen the role of CCDC51 in cardioprotection (e.g. [28,29,30,31]). We are not aware of any cardiac abnormalities in our patient. Detailed ocular phenotyping in mice lacking CCDC51 was not performed.

### 2.4. Expression Results of CCDC51, a Novel Candidate Gene Defect Underlying Mild arRCD

To further investigate the potential role of CCDC51, alias MITOK, in the retina, never described before, expression studies were performed. Transcriptomic analysis in public database revealed that *CCDC51* is ubiquitously expressed e.g., in the adrenal gland, blood, bone, brain, intestine, kidney, muscle, ovary, pancreas, skin, testis, but also eye (Hs.187657, found in former available UniGene database). This agrees with studies from others showing the presence of RNA and protein of MITOK in all tissues in humans and mice [27,32,33,34].

In addition, here, retinal expression was investigated using available human and mouse transcriptomic databases. Indeed, the human transcript of *CCDC51* and more specifically the four exons of *CCDC51* were found to be expressed in human retina [20] (Figure 2b). Expression of *CCDC51* was experimentally validated in human retina, in human universal tissues control cDNA, human fibroblasts, human blood, COS-1, and HeLa cells. A transcript for all tested tissues could be visualized by agarose gel electrophoresis at the expected size of ~660 bp (Figure 2c). The sequences of the human *CCDC51* transcripts were identical to the reference cDNA sequence of *CCDC51* (NM_001256964.1) with only one heterozygous synonymous variant present in universal tissues and fibroblasts compared to the reference sequence (Table S3). The *CCDC51* transcript of COS-1 cells showed thirteen homozygous nucleotide exchanges when compared to the human reference cDNA (Table S4). However, the respective sequences are present in the Chlorocebus sabaeus green monkey genome, which represents the origin of COS-1 cells.

**Figure 3:**
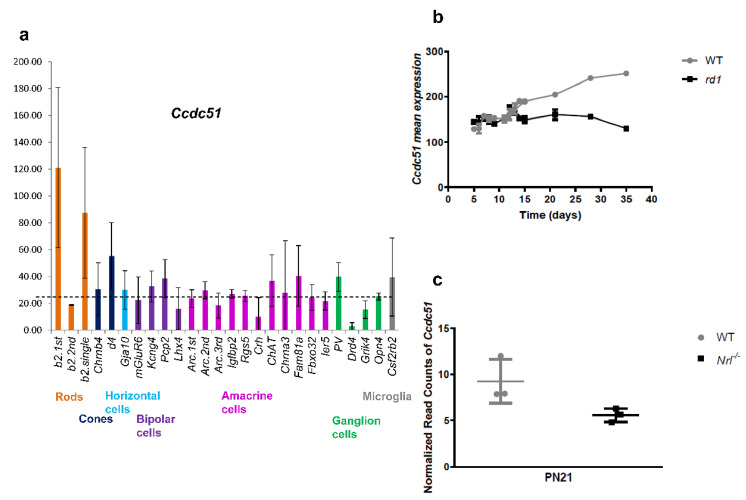
*CCDC51* analyses in mouse transcriptomic databases. (**a**) *Ccdc51* transcripts in six different cell types from mouse adult retina. The graph presents *Ccdc51* normalized expression values [36]. (**b**) *Ccdc51* expression in *rd1* and wild-type mice during retinal degeneration [37]. With photoreceptor degeneration from postnatal day (PN) 12 onwards, the *Ccdc51* expression decreases in the *rd1* mouse, which indirectly supports rod photoreceptor expression of *Ccdc51*. (**c**) *Ccdc51* expression in the rod-less mouse *Nrl*^−/−^. At PN day 21, *Ccdc51* transcript is less abundant in *Nrl*^−/−^ [38,39,40,41] compared to wild-type mice indicating indirectly *Ccdc51* expression in both rods and cones.

Interestingly, in mice, *Ccdc51* showed high expression in rod and possibly cone photoreceptors of the retinal-cell-type comparative transcriptome atlas [35,36] (Figure 3a). Due to high variability of the experiments for this gene in this database, it is unclear if *Ccdc51* is also expressed in horizontal, bipolar, amacrine, ganglion, and microglial cells (Figure 3a). Indirectly, expression of *Ccdc51* was also suggested in photoreceptors in the mouse model, *rd1*, where its relative expression decreases with age-dependent rod photoreceptor degeneration (Figure 3b) and in the rod-less mouse, *Nrl*^−/−^ at postnatal day 21, where the *Ccdc51* transcript is less abundant compared to wild-type mice (Figure 3c).

### 2.5. Detection of Endogenous CCDC51 in Human Cell Lines

**Figure 4:**
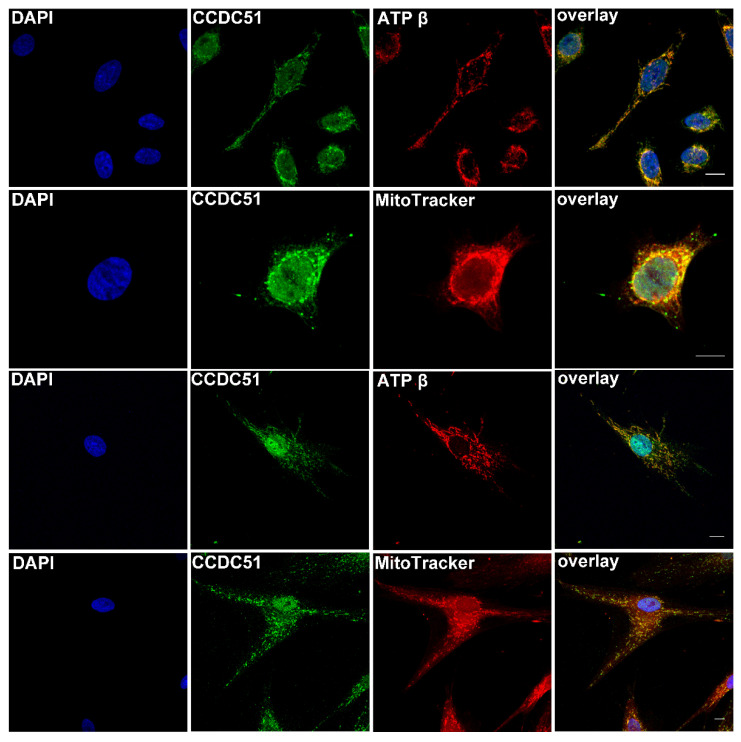
Detection of endogenous CCDC51/MITOK in human cell lines. Endogenous CCDC51/MITOK was detected with the rabbit anti-CCDC51 antibody in HeLa cells (**upper** two rows) and fibroblast cells (**lower** two rows) (green and yellow). A similar staining was obtained with the mitochondrial mouse anti-ATP synthase subunit beta antibody and a MitoTracker (red and yellow). Nuclei were stained with DAPI (blue) (bar = 20 μm).

Immunolocalization studies detected endogenous CCDC51, alias MITOK, protein in human fibroblast and HeLa cells (Figure 4, green) using fluorescent secondary antibodies. This staining partially overlapped (Figure 4, yellow) with the one obtained for a mitochondrial marker, using the anti-ATP synthase subunit beta monoclonal antibody (Figure 4 red) and a mitochondrion-selective probe (Figure 4 red), confirming that CCDC51/MITOK is a mitochondrial protein, as previously shown [27]. In addition, endogenous CCDC51 also locates in the nucleus, a finding which was previously described for U-2 OS cells (Human Bone Osteosarcoma Epithelial Cells) (https://www.proteinatlas.org/humancell/mitochondria, accessed on 9 April 2021).

### 2.6. Detection of CCDC51/MITOK in Non-Human Primate, Human, and Mouse Retina

**Figure 5:**
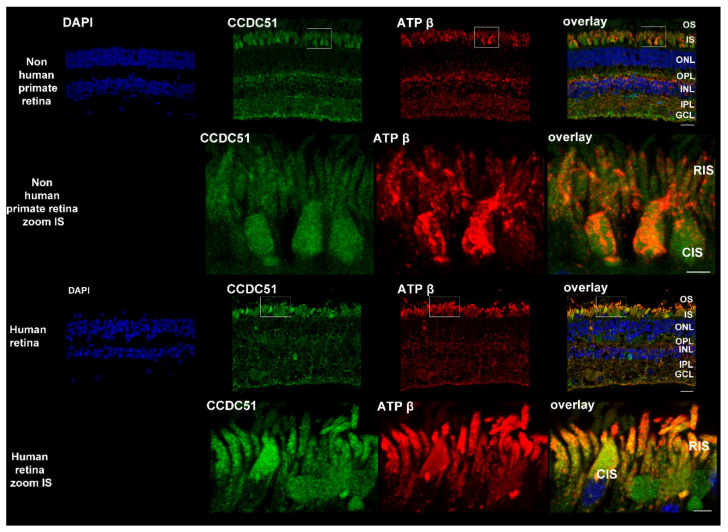
Detection of CCDC51 in the retina of non-human primate and human. Top: A non-human primate retinal section showed CCDC51 immunolocalization with the anti-CCDC51 antibody in different retinal cell layers (green and yellow). A similar staining with the mitochondrial mouse anti-ATP synthase subunit beta antibody (red and overlay) was obtained (bar = 20 μm). The enlarged image showed that the CCDC51 and ATP synthase subunit beta staining was found in close proximity, with some overlap in the inner segment of both rod and cone photoreceptor cells (zoom, green, and red and yellow, respectively). Nuclei were stained with DAPI (blue) (bar = 5 μm). Middle: A human retinal section showed CCDC51 immunolocalization with the validated rabbit anti-CCDC51 antibody in different retinal layers (green and yellow). A similar staining with the mitochondrial mouse anti-ATP synthase subunit beta antibody (red and overlay) was obtained (bar = 20 μm). The enlarged images showed that the CCDC51 and ATP synthase subunit beta staining was found in close proximity, with some overlap in the inner segment of both rod and cone photoreceptor cells (zoom, green, red, and yellow, respectively). Nuclei were stained with DAPI (blue) (bar = 5 μm). OS = outer segment, IS = inner segment, CIS = cone inner segment, RIS = rod inner segment, ONL = outer nuclear layer, OPL = outer plexiform layer, INL = inner nuclear layer, IPL = inner plexiform layer, GCL = ganglion cell layer.

**Figure 6:**
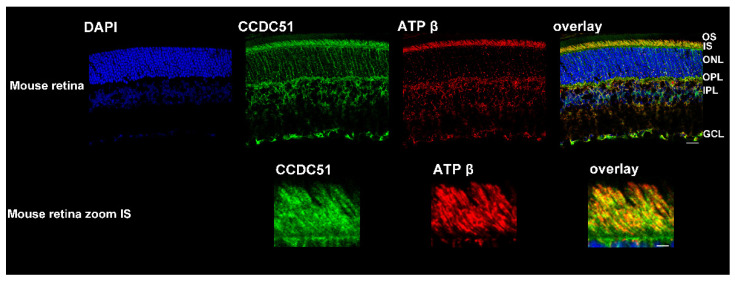
Detection of CCDC51 in mouse retina. Top: A mouse retinal section showed CCDC51 immunolocalization with the anti-CCDC51 antibody in different retinal cell layers (green and yellow). A similar staining with the mitochondrial mouse anti-ATP synthase subunit beta antibody (red and yellow) was obtained (bar = 20 μm). The enlarged images showed that the CCDC51 and ATP synthase subunit beta staining was found in close proximity, with some overlap in the inner segment of both rod and cone photoreceptor cells (zoom, green and red, respectively, and yellow). Nuclei were stained with DAPI (blue) (bar = 3 μm).

A non-human primate, a human, and a mouse retinal1 section revealed CCDC51, alias MITOK, immunolocalization by fluorescent staining in distinct retinal layers (Figure 5 and Figure 6 top, green, and overlay). A similar staining with the mitochondrial mouse anti-ATP synthase subunit beta antibody (Figure 5 and Figure 6 top, red, and overlay) was obtained. More specifically the CCDC51 and ATP synthase subunit beta staining was found in close vicinity and some colocalization in the inner segments of both rod and cone photoreceptor cells (Figure 5 and Figure 6 top, zoom, green and red and yellow, respectively, and overlay). Similarly, CCDC51 showed strong immunolocalization in the inner segments of photoreceptors in the human retina using immunohistochemical methods using horseradish peroxidase (Supplementary Figure S1). While a single band presumably representing endogenous and overexpressing CCDC51 in COS-1 and human fibroblast cells at the expected size of 46 kDa was visualized upon Western blot analyses, two bands with slightly distinct molecular weights were found in mouse and human retina (Supplementary Figure S2). The second band might be to a second isoform of CCDC51 present in the retina but not in other mammalian cells. The existence of a novel longer isoform of CCDC51 coding for a protein with a molecular weight of ~47 kDa is predicted (XP_011532415), which could be present in the upper band seen in the Western blot. Based on this, the mutation identified in the patient described herein could lead either to a truncated protein (c.303_305delinsGTGGAGATATGAAGATA p.(Trp103Argfs*25)) or to the absence of it secondary to nonsense-mediated mRNA decay. 

Together these findings confirmed that CCDC51, alias MITOK, also localizes in the mitochondria within the retina and that a defect of mitochondrial function due to mutations in CCDC51 is a possible cause of RCD observed in the investigated family.

## 3. Discussion

*CCDC51* encodes the coiled-coil domain-containing protein 51, also called MITOK. Mutations in this gene have never been described as disease causing. The only report we found was on a patient with learning difficulties, white matter abnormalities, elevated serum creatine kinase, oral-motor dyspraxia, facial hypotonia, and little involvement of other muscles who revealed a heterozygous 2 Mb deletion on chromosome 3, which encompasses, in addition to *DAG1*, the *CCDC51* locus as well. However, although the authors could not exclude effects from all genes they speculated that the heterozygous *DAG1* null mutation contributes to the severity of the phenotype [42]. In the family described herein, only the index patient (CIC00834) with bi-allelic *CCDC51* mutations is affected with RCD. None of the family members with heterozygous variants in *CCDC51* showed any clinical phenotype, indicating that mutations in *CCDC51* are causing autosomal recessive RCD.

Reported first in the rat brain [43], *CCDC51* is highly conserved across many species, from human to *Caenorhabditis elegans*. Exons 2–4 are predicted to code for a mitochondrial protein [26] of 411 amino acids (aa) with a predicted N-terminal mitochondrial targeting sequence, a coiled-coil domain (aa 111–173) and two transmembrane helical domains (aa TM1 202–222 and TM2 387–407). Our immunolocalization studies show that endogenous CCDC51 localizes in close proximity to mitochondrial markers (ATP synthase subunit beta and MitoTracker Probe) in human HeLa and fibroblast cells, which agrees with the predications of protein function. These findings are also in accordance with a recently published study that describes CCDC51/MITOK as a mitochondrial protein [27]. In addition, human and mouse CCDC51 was found in close proximity of the mitochondrial marker ATP synthase subunit beta in the retina and more specifically in the inner segments of rod and cone photoreceptors, compartments of the retina well known to harbor mitochondria.

**Figure 7:**
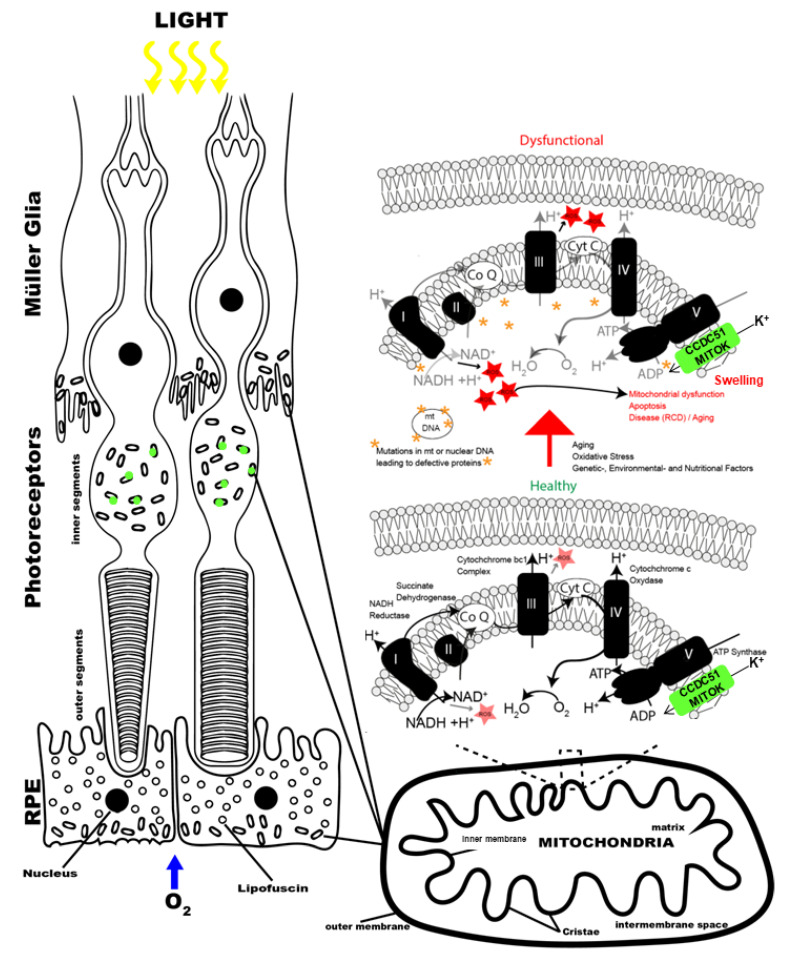
Schematic drawing of mitochondrial proteins in photoreceptor cells. (**Left**) outer retinal layers with mitochondria present in Müller glia, inner segment of photoreceptors, and retinal pigment epithelium. (**Right**) enlarged drawing of molecules implicated in mitochondrial function in healthy (bottom) and dysfunctional (top) conditions. The latter situation can be caused by mutations in mitochondrial or nuclear genes. (Figure adapted from [44]).

Mitochondria are important intracellular organelles as they provide the major source of cellular energy through oxidative phosphorylation, thereby delivering most of the adenosine triphosphate (ATP) requirements of eukaryotic cells [41,42,43]. They are present in every tissue, including the retina (specifically described for mitochondria present in the retina, Figure 7). These organelles have an outer and inner membrane, which form an intermembrane and an internal matrix space [42,43]. Mitochondrial oxidative phosphorylation resides in the inner mitochondrial membrane, which forms cristae (invaginations) providing an increased surface to generate ATP. The electron transport chain in the inner membrane is the site of oxidative phosphorylation and consists of five complexes of which complexes I–IV oxidize NADH and FADH_2_ through a controlled series of redox reactions, while complex V phosphorylates ADP to ATP. Ubiquinone, also known as coenzyme Q (Co Q), and cytochrome complex (cyt C) are cofactors of the electron transport chain that act as electron shuttles and importantly contribute to the mitochondrial respiratory chain function [44]. Defects in these complexes and/or cofactors lead to diminished mitochondrial ATP production and leakage, leading to an increased generation of reactive oxygen species (ROS) [45]. In addition to their function as ATP producers, mitochondria play an important role in preserving cell integrity and survival, by detoxification of ROS, mitochondrial dynamics (fission and fusion), the regulation of calcium homeostasis, nucleotide metabolism, and the biosynthesis of amino acids, cholesterol, and phospholipids [46,47]. Upon the onset a cellular energy crisis, mitochondrial function tends to decline. This is due to alternating inner membrane potential, imbalanced transmembrane ion transport, and an overproduction of free radicals, among other factors [28]. In such a situation, mitochondrial ATP-sensitive potassium (mitoK(ATP)) channels, which are channels consisting of a pore-forming subunit and by an ATP-binding subunit (sulphonylurea receptor) [29,30] open and close to regulate both internal Ca^2+^ concentration and the degree of membrane swelling. This helps restore proper membrane potential, allowing further H^+^ outflow, which continues to provide the proton gradient necessary for mitochondrial ATP synthesis. More specifically, K(ATP) channels are important for the electrophoretic transport of potassium ions (K^+^) inside the organelle matrix, which is inhibited by the physiological levels of ATP. Without aid from the potassium channels, the depletion of high energy phosphate would outpace the rate at which ATP could be created against an unfavorable electrochemical gradient [31].

Proteins that are important for the respiratory chain in the mitochondria are encoded by both mitochondrial DNA (mtDNA) and nuclear DNA (nDNA), indicating the importance of both genomes in the structure and function of the mitochondrial respiratory complexes [45]. Cells in highly metabolically active tissues such as the liver, kidney, the cardiac conductive system, and the eye strongly depend on ATP and have high numbers of mitochondria. Consequently, these tissues are susceptible to the defective mitochondrial energy output seen in mitochondrial disease [45]. Concerning the eye, mitochondria are located in the RPE, inner segments of photoreceptor cells, and Müller glial cells [44] (Figure 1 in [44] and Figure 7 here). Mutations in mtDNA and nuclear DNA affecting mitochondrial genes have been implicated in different retinal disorders (https://sph.uth.edu/retnet/disease.htm, accessed on 9 April 2021) (Table S5). In most cases the reported phenotypes differ from that seen in our patient. We are only aware of one gene defect in *IDH3B*, which leads to a similar non-syndromic RCD in three described families due to loss-of-function mutations [25,26]. However, after its initial description in 2008, it was also mentioned to be mutated in a patient with an unspecified syndrome [27]. *IDH3B* codes for the isocitrate dehydrogenase 3, beta subunit which catalyzes the oxidative decarboxylation of isocitrate to produce α-ketoglutarate, while converting nicotine adenine dinucleotide (NAD+) to NADH in the mitochondria. Functional analysis of two mutations resulted in substantial reductions in IDH3B activity and thus presumed mitochondrial dysfunction in the retina [23].

As mentioned before, CCDC51, alias MITOK, represents the pore-forming subunit of mitoK(ATP) channels. Recent findings showed that the absence of the pore subunit MITOK leads to a mitochondrial defect, namely instability in the mitochondrial membrane potential, widening the intracristae space and decreasing oxidative phosphorylation in vitro [27]. Similarly, the patient described herein carried a loss-of-function mutation, predicted to lead to an absence of a functional protein. It would be interesting to study how this mitochondrial gene defect leads exactly to photoreceptor degeneration in patients. Further studies using a mouse model may shine light on this [27]. However, often a relatively mild phenotype in RCD patients is not reproduced in mouse models [48,49,50,51]. Studies on fibroblasts or retinal cells from patient’s derived induced pluripotent stem cells [52] would be informative to evaluate putative effects of the *CCDC51* variant on mitochondrial function in the retina. Patient derived cells and organoids may display ultrastructural changes as previously reported in fibroblasts [53] or reveal mitochondrial dysfunction as altered oxygen consumption rates of live cells which could be measured with a kit (Agilent Seahorse Analytics, Courtaboeuf, Les Ulis, France).

Taken together, *CCDC51* is an excellent novel candidate gene, important for mitochondrial function underlying non-progressive RCD. Recent publications and the study described herein support this notion, despite the fact that variants seem to be ultra-rare and the causality in the retina needs to be further elucidated. Since gene defects leading to non-progressive IRD can also be implicated in syndromic forms, including genes important for mitochondrial function like *IDH3B* [23,24], future studies may identify disease-causing variants in syndromic forms of RCD.

## 4. Materials and Methods

### 4.1. Clinical Examination

The sporadic case affected with RCD (Figure 1, CIC00834, III.1) was clinically investigated at the national reference center for rare diseases REFERET of the Centre Hospitalier National d’Ophtalmologie des Quinze-Vingts as previously described [54].

### 4.2. Genetic Analyses

Blood samples of the index (CIC00834, III.1), the brother (CIC04408, III.2), the father (CIC04840, II.1), the paternal grandparents (CIC04405, I.1 and CIC04405, I.2), and the maternal grandmother (CIC04407, I.4) were collected for genetic research and genomic DNA was extracted as previously reported [55] (Figure 1). Research procedures adhered to the tenets of the Declaration of Helsinki and were approved by the local Ethics Committee (CPP, Ile de France V). Prior to genetic testing, informed consent was obtained from each study participant. Targeted NGS and WES were performed in collaboration with a company (IntegraGen, Evry, France) [11,13]. A panel of 123 genes known to be associated with retinal dystrophies was used for targeted NGS as previously described [11]. Array comparative genomic hybridization (CGH) was performed on the index patient (Figure 1, CIC00834, III.1), as before [56]. Subsequently, WES was performed in the index (Figure 1, CIC00834, III.1), in the brother (Figure 1, CIC04408, III.2), and father (Figure 1, CIC04840, II.1). Exons of DNA samples were captured and investigated as shown before with in-solution enrichment methodology (SureSelect Clinical Research Exome, Agilent, Massy, France) and NGS (Illumina HISEQ, Illumina, San Diego, CA, USA) [14]. For all subjects, overall WES coverage was at least 95% for a 10× and 87% for a 25× depth of coverage, respectively resulting in a mean sequencing depth of at least 73× per base (Tables S6–S8). Image analysis and base calling were performed with Real-Time Analysis software (Illumina) [15]. Genetic variation annotations were realized by an in-house pipeline (IntegraGen), and results were provided per sample or family in tabulated text files. Stringent filtering criteria were used to select the most likely pathogenic variant(s) as previously described [13]. Only variants showing a minor allele frequency of ≤0.5% in Exome Variants Server (EVS, http://evs.gs.washington.edu/EVS, accessed on 9 April 2021), HapMap (http://hapmap.ncbi.nlm.nih.gov, accessed on 9 April 2021), 1000Genomes (http://www.1000genomes.org, accessed on 9 April 2021), Exome Aggregation Consortium (ExAC, http://exac.broadinstitute.org, accessed on 9 April 2021), and genome Aggregation Database (GnomAD, http://gnomad.broadinstitute.org, accessed on 9 April 2021), in these databases were kept. The variants had to be nonsense, missense, splice site variants, or represent a small InDels. Variant pathogenicity was predicted with bioinformatic tools: Polymorphism Phenotyping v2 (PolyPhen2, http://genetics.bwh.harvard.edu/pph2, accessed on 9 April 2021), Sorting Intolerant From Tolerant (SIFT, http://sift.jcvi.org, accessed on 9 April 2021), MutationTaster (http://www.mutationtaster.org, accessed on 9 April 2021), and amino acid conservation across species with UCSC Genome Browser (http://genome.ucsc.edu/index.html, accessed on 9 April 2021; Human GRCh37/hg19 Assembly). Only variants predicted to be disease causing by at least one of the two programs, PolyPhen2 or SIFT, were analyzed further. In addition, the pathogenic character was predicted according to the American College of Medical Genetics and Genomics standards [21,22]. To validate variants detected by NGS and screen the whole candidate gene, Sanger sequencing was applied (conditions and oligomers will be provided on request). To detect putative CNVs in patients with monoallelic variants in the candidate gene, quantitative PCR experiments using primers covering all exons (Table S2) and a kit (SYBR Green Real-Time PCR Master Mixes, Applied Biosystems, Thermo Fischer, Villebon-sur-Yvette, France) at 60 °C annealing temperature was done. PCR reactions were performed in triplicates using two different amounts of genomic DNA (50 ng and 5 ng, in 5 μL) in a final volume of 20 μL, including 0.4 μL of each primer (0.2 μM final concentration), 4.2 μL of nuclease free water, and 10 μL of power SYBR Green PCR master mix. Positive and negative (no DNA template) controls were used in each run. PCR amplification was performed in a 96-well plate format on a 7500 Fast Real-Time PCR machine (Applied Biosystems) using the following conditions: 2 min at 50 °C, 10 min at 95 °C, 40 cycles (15 s at 95 °C and 1 min at 60 °C), 15 s at 95 °C, 1 min at 60 °C, and 15 s at 95 °C. The final dissociation step was included at the end of the PCR program to generate melting curves and assess primer specificity. Relative quantification was performed to normalize the number of *CCDC51* gene copies to those of the housekeeping gene GAPDH.

### 4.3. Gene Expression Analyses

Putative candidate genes showing putative disease-causing variants were investigated for their expression in different transcriptomic databases including UniGene (http://www.ncbi.nlm.nih.gov/unigene, accessed on 9 April 2021), the retinal gene expression profile database provided by Farkas and colleagues for human retina [20]; the in-house *rd1* mouse expression database (http://kbass.institut-vision.org/KBaSS/transcriptomics/index.php, accessed on 9 April 2021) to investigate if the gene is expressed in rod photoreceptors as these mice undergo early rod photoreceptor degeneration due to mutations in *Pde6b* [37]; a database based on transgenic mice, to see in which kind of retinal cells the candidate gene is expressed (https://www.fmi.ch/roska.data/index.php, accessed on 9 April 2021) [35,36], and the database of the “rod-less” *Nrl*^−/−^ mouse (https://retseq.nei.nih.gov, accessed on 9 April 2021) [57] to acquire knowledge if the candidate gene is also expressed in cone photoreceptors. Expression of the candidate gene has been experimentally validated in human retina (Clonetech, Palo Alto, California), in human control cDNA (universal, multi-tissues, Clonetech), in human fibroblast, in blood, in COS-1 and HeLa cells with primers in exon 2 and 4 of *CCDC51* (Ex2cDNAF: 5′-CACAGCATTCAGCAACGAGC-3′ and Ex4aR: 5′-GGACTACCTGCCTGTGACC-3′), and the sequence of the transcripts was validated by Sanger sequencing with the same primers (detailed conditions will be delivered on request).

### 4.4. Protein Localization Studies

The following online sites were used to predict the protein domains and protein localization of the candidate protein, MitoMiner 4.0, a database of mammalian mitochondrial localization evidence, phenotypes, and diseases (http://mitominer.mrc-mbu.cam.ac.uk/release-4.0/report.do?id=1003774, accessed on 9 April 2021), (https://www.proteinatlas.org/humancell/mitochondria, accessed on 9 April 2021) and (http://www.uniprot.org/uniprot/Q96ER9, accessed on 9 April 2021).

### 4.5. Specificity of Antibody against CCDC51

To perform functional analysis in mammalian cell lines and retinal tissues a rabbit anti-CCDC51 (HPA010980, Sigma, Saint Quentin Fallavier, France) antibody, directed against the human protein, was validated for its ability to detect CCDC51 in COS-1 cells overexpressing c-myc-tagged human *CCDC51* (GeneCust, Dubelange, Luxembourg) as previously described for other candidates [58,59] using the rabbit anti-CCDC51 (1:100) and mouse anti-c-myc (1:500) (11667149001, Roche, Basel, Switzerland) antibodies and with secondary donkey anti-rabbit conjugated with Alexa Fluor 488 (green, 1:1000) (711-545-152, Jackson Immuno Research Laboratories, Baltimore, MD, USA) and donkey anti-mouse with Cy3 (red, 1:1000) (715-165-150, Jackson Immuno Research) antibodies. Nuclei were stained with DAPI (1:1000) and cells were mounted with a mounting medium (Fluoromount-G, SouthernBiotech, Birmingham, AL, USA) using coverslips. Preparations were visualized with a fluorescent microscope (DM6000 B, Leica, Wetzlar, Germany) (Supplementary Figure S3). The signal obtained with anti-CCDC51 and anti-c-myc antibody overlapped showing a punctuate staining of the transfected cells. These findings indicate that, at least in this system, the antibody detects human CCDC51. Untransfected cells revealed no staining when tested with the anti-c-myc antibody and a faint staining when stained with anti-CCDC51 antibody in CCDC51 overexpressing COS-1 cells. The faint staining is most likely due to endogenous CCDC51 present in these cells. These findings were confirmed by Western blot analyses (Supplementary Figure S2). CCDC51 was detected in CCDC51-c-myc COS-1 transfected cells at the expected size of ~45 kDa. Similarly, endogenous CCDC51 was also detected in untransfected COS-1 cells. As a control, the same protein extracts were stained with anti-c-myc antibody revealing a specific band at the same size, only in transfected cells. Human fibroblast cells also revealed one specific band at the expected size of ~45 kDa on Western blot analysis. In contrast, mouse and human retina showed two bands with a slightly different molecular weight. Indeed, during the writing of the manuscript based on existing ESTs a CCDC51 protein containing 431 amino acids (XP_011532415) instead of 411 amino acids was predicted. The estimated size of this protein would be 47 kDa, which could represent a second isoform present in mouse and human retina (Supplementary Figure S2). Both isoforms differ in the 5′-region. The human anti-CCDC-51 antibody used for this work is directed against a common peptide of both isoforms. It represents a polyclonal antibody directed against the following amino acids: GLNEVREAQGKVTEAEKVFMVARGLVREAREDLEVHQAKLKEVRDRLDRVSREDSQYLELATLEHRMLQEEKRLRTAYLRAEDSEREKFSLFSAAVRESHEKERTRAERTKNWSLIGSVLGALIGVAGS. Indeed, 128 of these 129 amino acids are identical in *Chlorocebus sabaeus* green monkey, indicating that if the protein is indeed made in COS-1 cells, the protein should be detected. During the drafting of this manuscript an article was published describing CCDC51 (there called MITOK) as a novel mitochondrial protein validating the same antibody in MITOK-knockout HeLa cell lines [27].

### 4.6. Immunolocalization Studies in Human Cells and Retinal Tissue Using Fluorescence Imaging

The rabbit anti-CCDC51 antibody (1:100, HPA010980, Sigma) was used to stain HeLa and human fibroblast cells. Cells were washed with PBS, fixed in 4% formaldehyde for 10 min, rewashed with PBS, and most of the time quenched with 50 mM NH_4_Cl in PBS for 10 min. Subsequently cells were permeabilized for 10 min with 0.1% Triton X-100 in PBS, washed with PBS and blocked in PBS containing 2% BSA for 1 h. Cells were then incubated with primary antibodies (the rabbit anti-CCDC51 antibody) for 3 h at room temperature and washed 3 times with 0.1% Triton X-100 in PBS. CCDC51 was detected with the secondary donkey anti-rabbit conjugated with Alexa Fluor 488 (1:1000 green) (711-545-152, Jackson Immuno Research Laboratories). Co-staining with the mitochondrial marker, the ATP synthase subunit beta monoclonal antibody (1:1000 red) (3D5AB, Thermo Fisher Scientific, Villebon-sur-Yvette, France) was performed and visualized with the secondary donkey anti-mouse Cy3 (red, 1:1000) (715-165-150, Jackson Immuno Research) antibody. In addition, cells were stained alive using a mitochondrion-selective probe (1:1000 red) (Mitotracker Probe, Molecular Probes, Thermo Fisher Scientific). Subsequently cells were fixed with 4% formaldehyde and proceeded as described before together with the rabbit anti-CCDC51 antibody, which was visualized with secondary donkey anti-rabbit conjugated with Alexa Fluor 488. Nuclei were stained with DAPI (1:1000) and cells were mounted with a mounting medium (Fluoromount-G, SouthernBiotech, Birmingham, AL, USA) using coverslips.

Similarly, non-human primate (12 µm sections), human, and mouse (20 µm sections) retinas postfixed with acetone were stained with the validated rabbit anti-CCDC51 antibody (HPA010980, (1:1000) Sigma) and co-stained with the mitochondrial mouse anti-ATP-synthase beta antibody (1:1000) or the mitochondrion-selective probe (1:1000 red) (MitoTracker Probe, Molecular Probes, Thermo Fisher Scientific), using the same secondary antibodies and conditions as described for the human cell lines. Retinae stained only with the secondary antibodies or the isotype antibody (Rabbit (DA1E) mAb IgG XP^®^ Isotype Control #3900, Cell Signaling, Ozyme, Saint-Cyr-L’École, France) served as negative controls. Immunostainings were visualized with confocal microscopy (Olympus FV1200, Rungis, France).

### 4.7. Immunohistochemistry in Human Retinal Tissue Using Horseradish Peroxidase

Human retinas (50 µm sections) were postfixed with acetone and endogenous peroxidase was inhibited with 0.3% H_2_O_2_ (H1009–100mL lot STBH1090, Sigma). Subsequently, the retina was incubated with the rabbit anti-CCDC51 antibody ((1:1000), HPA010980, Sigma) and the secondary horseradish peroxidase antibody ((1.500) HTP goat anti-rabbit IgG(H + L), Jackson Immuno Research). The labeling was performed with a substrate (VIP peroxidase, SK-4600, Vector Laboratories, Inc., Burlingame, CA, USA) and images taken with a microscope (Carl Zeiss Microscopy, imager.M1, Jena, Germany).

## Figures and Tables

**Table 1:** Variants found to be homozygous in the affected girl CIC00843 co-segregates with the phenotype.

Chromosome	Gene Name	Refseq and MIM#	Variant	Minor Allele Frequency	Retinal Expression [20]
3	*CCDC51*	NM_001256964.1 No MIM#	c.244_246delinsGTGGAGATATGAAGATA p.(Trp82Valfs*4)	none reported	Yes

## Data Availability

The data that support the findings of this study are available from the corresponding author upon reasonable request.

## Supplementary Materials

The following are available online at https://www.mdpi.com/article/10.3390/ijms22157875/s1.
