# Supplementary material for: Mutated CCDC51 Coding for a Mitochondrial Protein, MITOK Is a Candidate Gene Defect for Autosomal Recessive Rod-Cone Dystrophy
Source: Int J Mol Sci. 2021 Jul 23;22(15):7875. doi: 10.3390/ijms22157875 (PMC8346125; doi:10.3390/ijms22157875)
Supplement: Supplementary file 1 [file ijms-22-07875-s001.zip › ijms-1218025-supplementary.pdf]

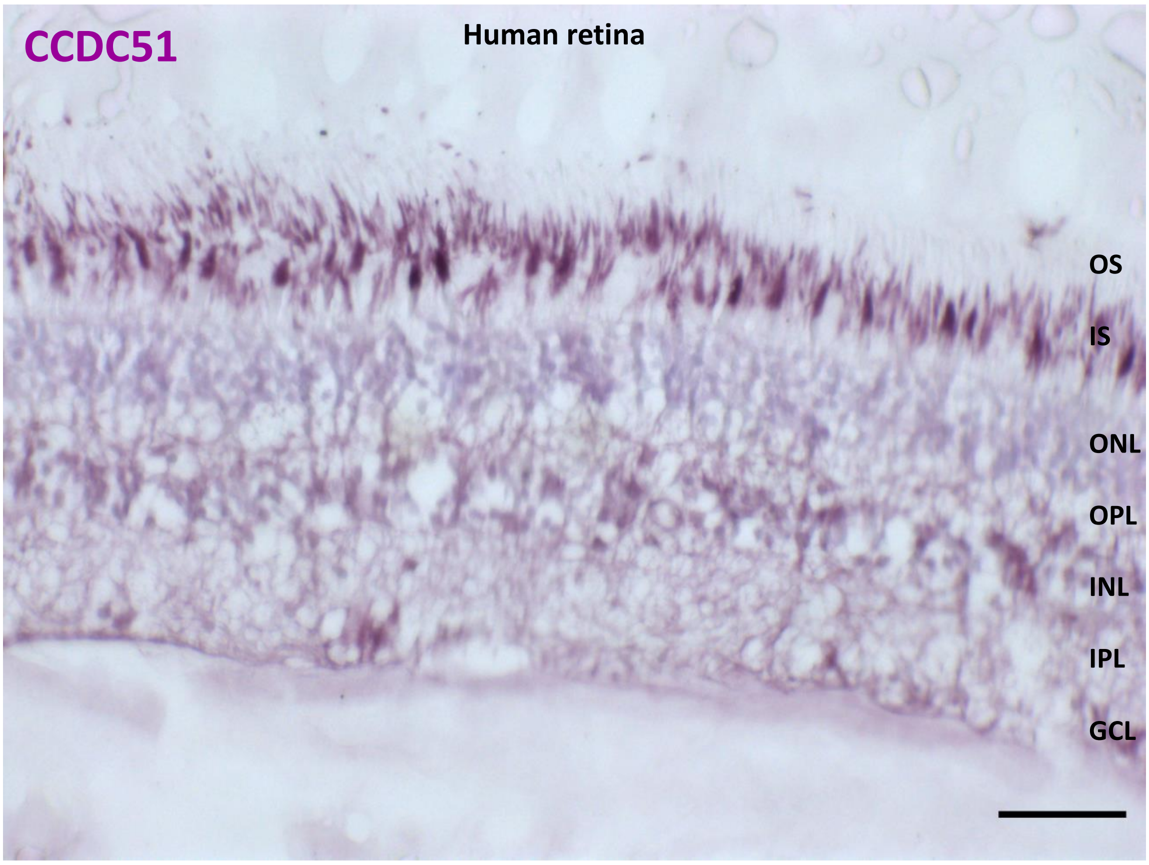

Figure S1: CCDC51 detection by immunochemistry with horseradish peroxidase on human retina.

CCDC51 was detected in the inner segments of the photoreceptors in the human retina using horseradish peroxidase labelling (bar = 50µm). OS = outer segments, IS = inner segments, ONL outer nuclear layer, OPL = outer plexiform layer, INL = inner nuclear layer; IPL = inner plexiform layer, GCL =ganglion cell layer.

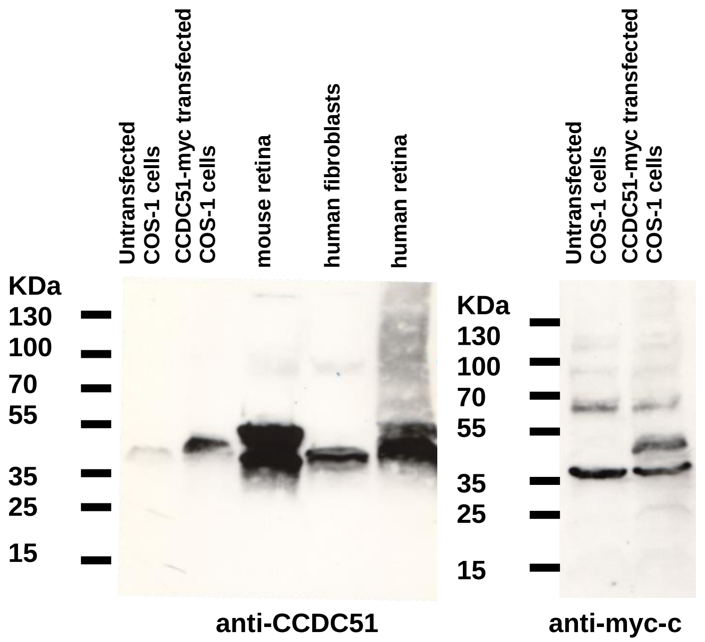

Figure S2: CCDC51 detection by Western blot analyses in cell lines, mouse and human retina.

CCDC51 was detected in CCDC51-c-myc COS-1 transfected cells at the expected size of ~45 kDa. At a lower amount, endogenous CCDC51 was also detected in untransfected COS-1 cells. As a control, the same protein extracts were stained with anti-c-myc antibody revealing a specific band at the same size, only in transfected cells. Human fibroblast cells reveal as well one specific band at the expected size of ~45 kDa. In contrast mouse and human retina reveal two bands with a slightly different molecular weight.

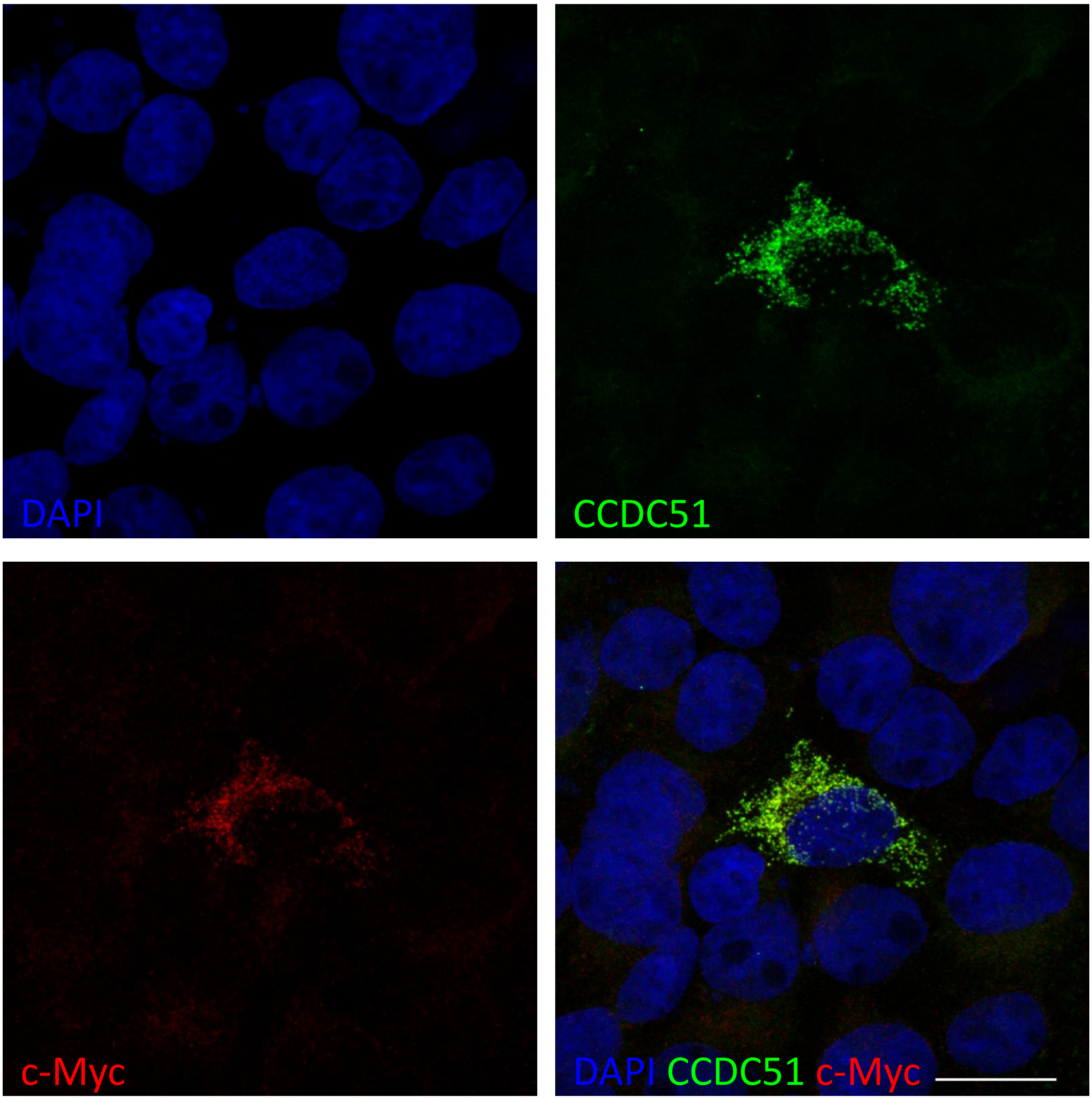

Figure S3: Antibody test on CCDC51 overexpressing COS-1 cells.

(a) The staining of the rabbit anti-CCDC51 (1:100) and the anti-c-myc (1:500) antibodies visualized with secondary donkey anti-rabbit conjugated with Alexa Fluor 488 (green, 1:1000) and donkey anti-mouse with Cy3 (red, 1:1000) antibodies with nuclei stained with DAPI (blue) visualized with a fluorescent microscope (DM6000 B, Leica, Wetzlar, Germany) (1:1000) overlapped in a dotted pattern (yellow) in COS-1 cells overexpressing CCDC51 (bar = 20µm).

S1 Table: *CCDC51* (NM_001256964.1) variants found heterozygous in patients with rod-cone dystrophy but with no second variant.

Patient #	Exon	Variant	Conservation	Minor Allele frequency	Polyphen 2	SIFT	Mutilation Tater	CNV by qPCR
CIC05511_F630	3	c.416G>A p.(Arg139His), rs752727840	97/97	ExAc: ALL:A=0.0028%-AFR:0.020%-AMR:0%-EAS:0%-SAS:0%-NFE:0.0027%-FIN:0.0045%-OTH:0%, ESP/EVS not found gnomAD: 7/246150, 0 homozygous 0.00002844	Probably damaging	Deleterious	Disease causing	No
CIC08015_F4473	IV3	c.477+16G>A p.?	10/54 show A in Bushbaby, Lesse Egyptian Jerboa, Chinese and Gold Hamster, mouse, rat, hedgehog, cape elephant shrew, lizard, stickleback and 1/54 C in armadillo	never reported	n.a.	n.a.	n.a.	No
CIC08507_F4807	4	c.508C>T, p.(Arg170Cys), rs146932588	44/100 different amino acids but never Cys	ExAC: ALL:T=0.0029%-AFR:0.0067%-AMR:0%-EAS:0%-SAS:0%-NFE:0.0055%-FIN:0%-OTH:0% ESP/EVS:EA: A=0.04% - AA: A=0.00% gnomAD: 8/274218, 0 homozygous, 0.00002917	Possibly damaging	Tolerated	Disease causing	No

S2 Table: *CCDC51* oligonucleotide primers used for quantitative PCR experiments

**Name**	**Oligonucleotide sequence**
CCDC51_ex1.1F	5’-CTGAGTGGCAGACGATTGGT-3’
CCDC51_ex1.1R	5’-CTGTCTACCTGCAGTGCTCT-3’
CCDC51_ex2-1F	5’-GTCCCCCTCACAGATCTCA-3’
CCDC51_ex2.1R	5’- CCACCTCCTCAGGTCTTTTC -3’
CCDC51_ex2cDNAF	5’-CACAGCATTCAGCAACGAGC-3’
CCDC51_ex2.2R	5’- CACCTCTGTCACCTTTCCCT -3’
CCDC51_ex3.1F	5’-CTCCGGTTTTCTCTTTGCAG-3’
CCDC51_ex3.1R	5’- GCTCGAGAGTAGCCAGTTCC -3’
CCDC51_ex4.1F	5’-CACCTTGTGCCCATGTAATC-3’
CCDC51_ex4.1R	5’-GCGCTCCTTCTCATGACTTT-3’
CCDC51_ex4.2F	5’- CCACAATCTCATGGTGGACTT -3'
CCDC51_ex4.2R	5’- ATGGACTTGCCTGGAATGAC -3'
CCDC51_ex4.3F	5’- GGTTCAGCTTGTAAAGTCTGC -3'
CCDC51_ex4.3R (4bR)	5’- GATGGTGTTCCTGTTGACTTG -3'
CCDC51_ex4.4F	5’- CCACACTGCCTGTGCTCTA -3'
CCDC51_ex4.4R	5’- GCTCCTTCAGATTGAGGTTG -3'
GAPDH_F_QPCR	5’- CTCCCCACACACATGCACTTA-3'
GAPDH_R_QPCR	5’- TTGCCAAGTTGCCTGTCCTT-3'

S3 Table: Variants found heterozygous in human retina, universal tissue and fibroblasts compared to human *CCDC51* (NM_001256964.1)

#	Nucleotide exchange	Amino acid exchange	Reference	Conclusion
1	c.805T>C	p.(Leu269Leu)	rs2279077 ExAc : ALL:C=37.83% -AFR:27.35%-AMR:22.64%-EAS:30.90%-SAS:32.50%-NFE:45.23%-FIN:39.17%-OTH:39.37% ESP: EA: G=46.56% - AA: G=26.92% gnomAD: 104672/276646 with 20920 homozygous Frequency: 0.3784	Polymorphism

S4 Table: Variants found homozygous in COS-1 cells compared to human *CCDC51* (NM_001256964.1)

#	Nucleotide exchange	Amino acid exchange	Reference	Nucleotide or amino acid exchange occurs in Chlorocebus sabaeus green monkey genome
1	c.770G>T	p.(Arg257Leu)	-	yes
2	c.810G>A	p.(Arg270Arg)	-	yes
4	c.735A>G	p.(Gln245Gln)	-	yes
5	c.711G>A	p.(Ala237Ala)	-	yes
6	c.696T>C	p.(Ala232Ala)	-	yes
7	c.678A>G	p.(Arg226Arg)	-	yes
8	c.675G>C	p.(Val225Val)	rs771909567 ExAc : ALL:C=0.00041 %-AFR:0%-AMR:0%-EAS:0.0058%-SAS:0%-NFE:0%-FIN:0%-OTH:0% gnomAD: 1/246098 with 0 homozygous Frequency: 0.000004063	yes
9	c.633G>A	p.(Gly211Gly)	-	yes
10	c.591T>G	p.(Ala197Ala)	-	yes
11	c.534G>A	p.(Lys178Lys)	-	yes

12	c.528A>C	p.(Arg176Arg)	-	yes
13	c.489G>A	p.(Arg163Arg)	-	yes

S5 Table: Mutations in mtDNA and nDNA mitochondrial genes leading to a retinal phenotype

S5.1 Table Mutations in mtDNA mitochondrial genes leading to a retinal phenotype

Symbols	Disease	Impact on mitochondrial proteins	References
KSS (MIM: 530000)	Kearns-Sayre syndrome, including retinal pigmentary degeneration and one or more of the following: cardiac conduction abnormality, cerebrospinal fluid abnormalities or cerebella dysfunction	Several mitochondrial proteins	[47, 48]
LHON (MIM: 535000)	Leber hereditary optic neuropathy	complex I, III or IV	[49-57]
MT-TL1, *DMDF*, *TRNL 1* (MIM: 520000 and 590050)	macular pattern dystrophy with type II diabetes and deafness	leucine tRNA 1 (UUA/G), nt 3230-3304	[58-62]
MT-ATP6, *ATP6*, *NARP* (MIM: 516060 and 551500)	retinitis pigmentosa with developmental and neurological abnormalities; Leigh syndrome; Leber hereditary optic neuropathy (developmental delay, neuropathy, ataxia and RP, with or without optic atrophy)	complex V ATPase 6 subunit, nt 8527-9207	[63-66]
MT-TH, *TRNH* (MIM: 590040)	pigmentary retinopathy and sensorineural hearing loss, cardiomyopathy	histidine tRNA, nt 12138-12206	[67]
MT-TS2, *TRNS2* (MIM: 500004 and 590085)	retinitis pigmentosa with progressive sensorineural hearing loss	tRNA 2 (AGU/C), nt 12207-12265	[68]
MT-TP, TRNP (MIM: 590075)	retinitis pigmentosa with deafness and	proline tRNA, nt 15955-16023	[69]

	neurological abnormalities, Parkinson disease and/or myopathy		

S5 1.2 Mutations in nDNA mitochondrial genes leading to a retinal phenotype

Symbols	Chromosomal localization	Disease	Impact on mitochondrial proteins	References
MFN2, *CMT6*, *CMT2A2*, *MARF* (MIM: 608507, 609260 and 601152)	1p36.22	dominant optic atrophy with neuropathy and myopathy; dominant Charcot-Marie-Tooth disease	mitofusin 2 is important for fusion of mitochondria and contributes to mitochondrial morphology and distribution	[70]
OPA1 (MIM: 125250, 165500 and 605290)	3q29	dominant optic atrophy, Kjer type; dominant optic atrophy with sensorineural hearing loss	OPA1 protein is a dynamin-related GTPase which localizes to mitochondria	[71-84]
WFS1, *DFNA38* (MIM: 222300 and 598500)	4p16.1	recessive, and dominant (low frequency) sensorineural hearing loss, include diabetes, optic atrophy, deafness, often associated with multiple mitochondrial deletions	Altered mitochondrial dynamics	[85-93]
SLC25A46, *CMT6B* and *HMSN6B* (MIM: 610826, 616505)	5q22.1	recessive syndromic optic atrophy, variable neurologic findings including cerebellar ataxia, motor and sensory neuropathy, and pontocerebellar hypoplasia; symptoms are consistent with	solute carrier family 25 membrane protein is an integral component of the mitochondrial outer membrane and participates in mitochondrial function and mitochondrial fusion	[94, 95]

		Charcot-Marie-Tooth disease and Leigh syndrome		
RTN4IP1, *OPA10* (MIM: 610502 and 616732)	6q21	recessive optic atrophy, non-syndromic and syndromic with or without further neurologic findings	reticulon 4 interacting protein is a mitochondrial ubiquinol oxydo-reductase protein important for the respiratory complex I and IV activities and involved in retinal ganglion cell function and neural-retinal development	[96]
TMEM126A, *OPA7* (MIM: 165500 and 612988, 612989	11q14	syndromic optic atrophy	TMEM126A is a mitochondrial located mRNA (MLR) protein of the mitochondrial inner membrane	[97-99]
C12orf65, *COXPD7*, *SPG55* (MIM: 613541, 613559 and 615035)	12q24	recessive spastic paraplegia, neuropathy and optic atrophy	chromosome 12 open reading frame 65 is a nuclear-encoded mitochondrial matrix protein involved in mitochondrial protein synthesis	[100, 101]
OPA8 (MIM: 616648)	16q21-q22.3	dominant optic atrophy with late-onset sensorineural hearing loss hearing loss, increased central conductance times and cardiac abnormalities, Kjer type	lack of functional OPA8 protein leads to subsarcolemmal accumulations of mitochondria and slight increase in mtDNA content	[102]
OPA3, MGA3 (MIM: 165300, 165500, 258501, 606580)	19q13.32	recessive optic atrophy with ataxia and 3-methylglutaconic aciduria; dominant optic atrophy with cataract, ataxia and	OPA3 plays a role in mitochondrial processes: Opa3 binds to prohibitin, a molecular chaperone with additional roles in	[103-109]

		areflexia, also called Costeff optic atrophy syndrome; symptoms related to 3-methylglutaconic aciduria include early-onset optic atrophy, cognitive deficit, extrapyramidal abnormalities, ataxia and spastic paraplegia	insulin/IGF-1 signaling and maintaining the integrity of the inner mitochondrial membrane. As a chaperone, prohibitin shuttles cardiolipin between the mitochondria and the nucleus; a form of inter-organelle communication that appears most important in tissues with high oxygen demands (e.g. the retinal pigment cells.	
IDH3B, RP46 (MIM: 268000, 604526, 612572)	20p13	Nonsyndromic recessive retinitis pigmentosa	NAD(+)-specific isocitrate dehydrogenase (3 beta isocitrate dehydrogenase) catalyzes conversion of isocitrate to α-ketogluterate in the citric acid cycle (Krebs cycle); the Krebs cycle, localized in mitochondria	[110]
TIMM8A, DDP, DDP2, DFN1(MIM: 300356, 304700 and 311150)	Xq22.a	optic atrophy with deafness-dystonia syndrome also known as Mohr-Tranebjaerg or Jensen syndrome	inner mitochondrial membrane translocase 8 homolog, protein involved in transport of metabolites into mitochondria	[111-113]

S6 Table: Coverage and read depth from whole exome sequencing for the affected girl, CIC00834.

Chr	Size of the target regions (bp)	1X Coverage (%)	4X Coverage (%)	10X Coverage (%)	25X Coverage (%)	Mean depth (X)
1	4738564	98.73	97.48	95.43	88.9	81.91
2	3471855	98.96	98	96.37	90.61	85.34
3	2767223	99.62	98.94	97.57	92.15	86.84
4	1871681	99.36	98.48	97.15	91.2	86.12
5	2168450	99.01	98.1	96.51	90.55	84.53
6	2470987	98.48	97.49	95.89	90.26	85.86
7	2280798	97.53	95.96	93.5	86.19	78.98
8	1642199	97.9	96.85	95.05	88.85	80.91
9	1891158	98.49	97.08	94.86	87.87	80.46
10	1873001	98	96.99	95.32	89.04	80.45
11	2832686	99.35	98.02	95.68	88.8	80.47
12	2618601	99.3	98.32	96.42	89.73	81.87
13	859203	99.4	98.61	97.19	91.27	82.55
14	1604688	99.51	98.57	96.74	90.29	81.4
15	1672882	97.22	95.95	93.95	87.68	79.94
16	2028152	97.22	95.34	92.47	84.47	75.65
17	2735968	98.57	96.9	94.1	86.29	76.74
18	741097	99.15	98.12	96.39	90.48	83.27
19	3044127	98.1	95.49	91.38	81.2	67.22
20	1171367	99.05	97.73	95.54	88.87	79.65
21	480492	99.26	98.21	96.11	89	78.65
22	1025109	97.94	95.9	92.83	84.67	73.34
X	1756723	94.98	94.17	92.84	87.73	82.02
Y	91440	1.19	0.64	0.45	0.31	0.16
Total	47838451	98.48	97.25	95.19	88.53	80.62

S7 Table: Coverage and read depth from whole exome sequencing for the unaffected brother, CIC04408.

Chr	Size of the target regions (bp)	1X Coverage (%)	4X Coverage (%)	10X Coverage (%)	25X Coverage (%)	Mean depth (X)
1	4738564	98.66	97.38	95.16	87.76	76.22
2	3471855	98.88	97.88	96.07	89.14	78.28
3	2767223	99.6	98.88	97.33	90.75	80.33
4	1871681	99.34	98.41	96.78	89.42	78.29
5	2168450	99.03	97.97	96.1	88.92	77.57
6	2470987	98.43	97.37	95.6	89	79.22
7	2280798	97.4	95.81	93.24	85.01	73.32
8	1642199	97.88	96.78	94.7	87.18	74.52
9	1891158	98.4	96.98	94.57	86.81	75.3
10	1873001	97.98	96.85	95	87.6	74.58
11	2832686	99.28	97.99	95.53	87.93	75.62
12	2618601	99.27	98.27	96.18	88.46	75.79
13	859203	99.31	98.53	96.94	89.58	75.13
14	1604688	99.43	98.54	96.49	88.88	75.32
15	1672882	97.1	95.8	93.65	86.39	74.6
16	2028152	97.21	95.24	92.28	83.76	71.92
17	2735968	98.56	96.87	93.97	85.44	72.87
18	741097	99.13	98.01	95.97	88.99	76.34
19	3044127	97.98	95.43	91.27	80.67	65.27
20	1171367	98.91	97.63	95.37	87.98	75.4
21	480492	99.36	98.09	95.69	87.59	73.81
22	1025109	97.85	95.85	92.7	84.06	70.34
X	1756723	94.56	93.02	88.88	71.11	38.34
Y	91440	57.49	55.35	51.25	35.67	20.47
Total	47838451	98.42	97.11	94.76	86.63	73.41

S8 Table: Coverage and read depth from whole exome sequencing for the unaffected parent, CIC04840.

Chr	Size of the target regions (bp)	1X Coverage (%)	4X Coverage (%)	10X Coverage (%)	25X Coverage (%)	Mean depth (X)
1	4738564	98.78	97.63	95.81	90.26	93.68
2	3471855	98.97	98.08	96.61	91.76	97.28
3	2767223	99.67	99.09	97.85	93.19	99.42
4	1871681	99.42	98.64	97.34	92.22	98.01
5	2168450	99.09	98.18	96.72	91.67	96.33
6	2470987	98.51	97.6	96.13	91.23	97.39
7	2280798	97.63	96.11	94	87.62	90.05
8	1642199	98.02	97	95.35	90.21	92.5
9	1891158	98.58	97.3	95.27	89.26	92.34
10	1873001	98.06	97.17	95.62	90.26	91.86
11	2832686	99.4	98.28	96.24	90.39	92.47
12	2618601	99.34	98.44	96.73	91.19	93.54
13	859203	99.43	98.74	97.41	92.5	93.86
14	1604688	99.52	98.66	97.01	91.58	92.87
15	1672882	97.2	96.07	94.25	88.77	91.42
16	2028152	97.36	95.57	93.04	86.18	87.08
17	2735968	98.66	97.15	94.69	87.98	88.42
18	741097	99.21	98.19	96.63	91.63	95.03
19	3044127	98.21	95.91	92.38	83.44	78.08
20	1171367	99.08	97.94	96.08	90.47	91.97
21	480492	99.42	98.44	96.62	90.2	90.13
22	1025109	98.1	96.3	93.56	86.67	84.72
X	1756723	94.72	93.48	90.38	77.1	47.62
Y	91440	57.7	55.69	52.67	41.2	25.62
Total	47838451	98.54	97.39	95.47	89.38	90.26
